# Prevention of airway hyperresponsiveness induced by left ventricular dysfunction in rats

**DOI:** 10.1186/1465-9921-13-114

**Published:** 2012-12-13

**Authors:** Ferenc Petak, Gergely Albu, Eniko Lele, Maurice Beghetti, Walid Habre

**Affiliations:** 1 Department of Medical Physics and Informatics, University of Szeged, 9 Koranyi fasor, H−6720, Szeged, Hungary; 2 Anaesthesiological Investigations Unit, University Hospitals of Geneva, 1 Rue Michel Servet, CH-1205, Geneva, Switzerland; 3 Paediatric Cardiology Unit, Department of Paediatrics, Geneva Children's Hospital, 6, Rue Willy Donze, CH-1205, Geneva, Switzerland; 4 Paediatric Anaesthesia Unit, Geneva Children’s Hospital, University Hospitals of Geneva, 6, Rue Willy Donze, CH-1205, Geneva, Switzerland

## Abstract

**Background:**

The effectiveness of strategies for treatment of the altered static lung volume and against the development of bronchial hyperreactivity (BHR) following a left ventricular dysfunction (LVD) induced by myocardial ischaemia was investigated in a rat model of sustained postcapillary pulmonary hypertension.

**Methods:**

Airway resistance (Raw) was identified from the respiratory system input impedance (Zrs) in four groups of rats. End-expiratory lung volume (EELV) was determined plethysmographically, and Zrs was measured under baseline conditions and following iv infusions of 2, 6 or 18 μg/kg/min methacholine. Sham surgery was performed in the rats in Group C, while the left interventricular coronary artery was ligated and Zrs and its changes following identical methacholine challenges were reassessed in the same rats 8 weeks later, during which no treatment was applied (Group I), or the animals were treated daily with a combination of an angiotensin enzyme converter inhibitor and a diuretic (enalapril and furosemide, Group IE), or a calcium channel blocker (diltiazem, Group ID). The equivalent dose of methacholine causing a 100% increase in Raw (ED_50_) was determined in each group. Diastolic pulmonary arterial pressure (Pap_D_) was assessed by introducing a catheter into the pulmonary artery.

**Results:**

The sustained presence of a LVD increased Pap_D_ in all groups of rats, with variable but significant elevations in Groups I (p = 0.004), ID (p = 0.013) and IE (p = 0.006). A LVD for 8 weeks induced no changes in baseline Raw but elevated the EELV independently of the treatments. In Group I, BHR consistently developed following the LVD, with a significant decrease in ED_50_ from 10.0 ± 2.5 to 6.9 ± 2.5 μg/kg/min (p = 0.006). The BHR was completely abolished in both Groups ID and IE, with no changes in ED_50_ (9.5 ± 3.6 vs. 10.7 ± 4.7, p = 0.33 and 10.6 ± 2.1 vs. 9.8 ± 3.5 μg/kg/min p = 0.56, respectively).

**Conclusions:**

These findings suggest that a LVD following coronary ischaemia consistently induces BHR. The more consistent efficacy of both treatment strategies in preventing BHR than in treating the adverse pulmonary vascular consequences suggests the benefit of both calcium channel blockade and ACE inhibition to counteract the airway susceptibility following a LVD.

## Background

The results of previous clinical and experimental studies clearly established that a left ventricular dysfunction (LVD) leads to a lung function impairment manifested in airflow limitation and compromised lung compliance [1,2]. There is also increasing evidence that the diminished airway function following a LVD results in the development of bronchial hyperreactivity (BHR) in response to exogenous constrictor stimuli [1,3-5]. The pulmonary congestion subsequent to chronic LVD in patients advances the development of clinical symptoms, such as wheezing, coughing, dyspnea and recurrent bronchospasm triggered by exposures to various provocation agonists [1,4]. A number of factors may contribute to the development of BHR following a LVD, including a decrease in airway cross-sectional area [6] due to the compression of the airways by the dilated pulmonary vessels [4], an elevated capillary hydrostatic pressure leading to mucosal swelling [1,3], and airway wall hypertrophy [7].

In clinical practice, different treatment strategies are considered in the presence of LVD in order to improve the cardiac output, to advance fluid clearance and to decrease pulmonary congestion. Angiotensin converting enzyme (ACE) inhibitors are commonly regarded as first-line therapy through which to counteract the renin-angiotensin pathway and hence the production and secretion of aldosterone [8], with an ultimate reduction of the systemic vascular resistance and relief of the vascular engorgement. Alternatively, there has been some interest in the blockade of calcium entry, which can potentially improve the left ventricular function via systemic arterial vasodilation, leading to a reduced ventricular afterload, reflex activation of the sympathetic nervous system and direct improvement of the myocardial inotropic depression [9,10]. Despite these well-established beneficial effects of these treatments on the haemodynamic outcomes, there have been no studies aimed at establishing how these treatment strategies ultimately alter the adverse pulmonary consequences of a LVD. Accordingly, the effectiveness of such treatments as concerns the alterations in the basal airway and tissue mechanical properties, lung volume and airway responsiveness has not been characterized.

We therefore set out to explore the pulmonary consequences of these common treatment strategies, applied in the presence of a sustained elevation in pulmonary venous pressure following the induction of a LVD in a well-established experimental model [11] mimicking the adverse pulmonary symptoms of chronic lung congestion. Changes in pulmonary haemodynamics, basal airway and tissue mechanics and lung responsiveness were characterized following a decrease in the insult from the vascular remodelling by i) an ACE inhibitor combined with a diuretic and ii) a calcium channel blocker.

## Methods

### Animal preparations

The experimental protocol was approved by the Experimental Ethics Committee of the University of Geneva (No. 09-45) and the Animal Welfare Committee of the Canton of Geneva (No. 1051/3542/3). On the experimental days, adult male Sprague–Dawley rats (initially weighing 342-395 g) were anaesthetized by the intraperitoneal injection of chloral hydrate (350 mg/kg). Tracheal intubation was achieved with a polyethylene cannula (16-gauge, Braun, Melsungen, Germany) and the rats were allowed to breathe spontaneously for the lung volume measurement. The rats were then mechanically ventilated with a tidal volume of 7 ml/kg body weight, a positive end-expiratory pressure of 2.5 cmH_2_O, and a respiratory rate of 60-70/min (constant volume-cycled rodent ventilator, model 683, Harvard Apparatus Co Inc., South Natick, MA, USA). After the completion of plethysmography, the femoral vein was cannulated and a continuous iv infusion of midazolam (1 mg/kg/h) with fentanyl (40 μg/kg/h) and atracurium (1 mg/kg/h) was administered to ensure an adequate anaesthesia and analgesia level throughout the study. The femoral artery was cannulated (Abocath 22 G) and attached to a pressure transducer (Model 156 PCE 06-GW2, Honeywell, Zürich, Switzerland) for continuous blood pressure monitoring. An arterial line was also used for blood gas analysis (Model 505, Acid Base Laboratory, Copenhagen, Denmark). The airway pressure, arterial pressure, ECG and rectal temperature were continuously monitored by a data collection and acquisition system (Biopac, Santa Barbara, CA, USA).

### Surgical preparations for the induction of LVD

An additional bolus of fentanyl was administered intra-arterially (15 μg/kg) to strengthen the level of analgesia. The heart in each rat was then surgically exposed by a left lateral thoracotomy (5^th^ intercostal). A prophylactic dose of lidocaine (2 mg/kg intra-arterially) was administered to prevent arrhythmias that may arise from ligation of the coronary artery. After opening of the pericardium, a silk thread was sutured around the proximal part of the descending interventricular coronary artery (next to the departure of the pulmonary artery).

### Treatments and protocol groups

In the animals assigned to Groups I (n = 10), ID (n = 9) and IE (n = 10), the knot was tightened to induce a LVD via myocardial ischaemia, while in the rats enrolled in Group C (n = 10), sham surgery was performed by removal of the silk thread without occlusion of the coronary artery. The thorax was closed after removing the residual air in the pleural cavity, and a long-acting local anaesthetic (bupivacaïne 0.25%, 0.8-1.2 ml) was infiltrated around the surgical wounds. 0.5 mg/kg of buprenorphine (Temgesic®) was injected subcutaneously before the rat was allowed to recover from the anaesthesia, and this dose was repeated 18 h later to ensure adequate analgesia.

**Figure 1:**
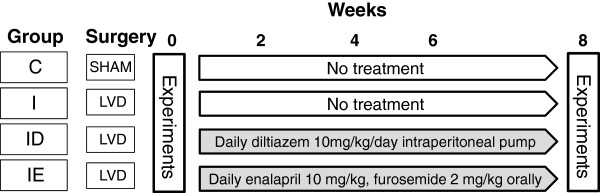
Scheme of the experimental protocol.

After creation of the LVD, the animals were randomized to be included to receive no further treatment during the 8-week study period (Group I), regular treatment with a calcium channel blocker (diltiazem 10 mg/kg/day via daily ip injection) for 8 weeks (Group ID), or regular treatment with an ACE inhibitor (enalapril, 10 mg/kg/day, via oral gavage) supplemented with a diuretic (furosemide 2 mg/kg/day, via oral gavage) until the next experimental day 8 weeks later (Group IE). The daily itraperitoneal and oral treatments were performed by gentle restraining of the animals without sedation. The route of administration of the treatment agents and the doses were chosen on the basis of their pharmacokinetic properties and the manufacturers’ recommendations. Eight weeks after the completion of the first part of the investigations, the animals were re-evaluated as detailed above, and the second part of the investigations was performed. The scheme of the experiments is presented in Figure 1.

### Measurements of end-expiratory lung volume (EELV)

After the anaesthesia with chloral hydrate and the surgical preparations, the measurements of EELV were performed in all groups by using a body plethysmograph, as detailed earlier [12]. Briefly, the trachea was occluded at end-expiration until 3 or 4 spontaneous inspiratory efforts had been generated by the animal in the closed box. The changes in tracheal pressure and plethysmograph box pressure during these manoeuvres were recorded, and EELV was calculated by applying Boyle's law to the relationship between the tracheal pressure and the box pressure after correction for the box impedance [13]. The recordings of box pressure were corrected for the thermal characteristics of the plethysmograph [13]; this minimized the biasing effects of the different breathing frequencies during the inspiratory efforts. The sufficient spontaneous breathing manoeuvres were required for the measurement of EELV were not always achieved in the anaesthetized animals: these measurements were successfully accomplished in 8, 7, 9 and 10 animals in Groups C, I, ID and IE, respectively.

### Forced oscillatory measurements

The contributions of the airway and tissue mechanical properties to the total respiratory resistance were assessed by the forced oscillation technique in the anesthetized animals through measurement of the mechanical impedance of the respiratory system (Zrs), as described in detail previously [14,15]. Briefly, the tracheal cannula was connected from the respirator to a loudspeaker-in-box system at end-expiration. Zrs was measured with the wave-tube technique by introducing pseudorandom forced oscillations at frequencies between 0.5 and 21 Hz at end-expiration. Two identical pressure transducers (Model 33NA002D, ICSensors, Malpitas, CA, USA) were used to measure the lateral pressures at the loudspeaker and at the tracheal end of the wave-tube, and Zrs was calculated as the load impedance of the wave-tube by using fast Fourier transformation [14].

To separate the airway and tissue mechanics, a model containing a frequency-independent resistance (Raw) and inertance (Iaw) in series with a constant-phase tissue model [16] including tissue damping (G) and elastance (H) was fitted to the Zrs spectra by minimizing the differences between the measured and modelled impedance values:

(1) $$\mathrm{Zrs}=\mathrm{Raw}+j\omega \mathrm{Iaw}+\left(G-\mathrm{jH}\right)/\omega^{\alpha }$$

where j is the imaginary unit, ω is the angular frequency (2πf) andα =2/π arctan(H/G). When this model is fitted to Zrs spectra, the parameter Raw is primarily related to the overall airway geometry, as the contribution of the chest wall to the frequency-independent Newtonian resistance is minor [17]. Similarly, the inertia of the gas in the airways predominates in the parameter Iaw [17]. The equivalent dose causing a 50% increase in Raw (ED_50_) was calculated in each rat by linear interpolation. Following these measurements, pulmonary arterial pressure was measured as described below. The specific airway resistance (SRaw) was calculated as Raw*EELV.

### Measurement of pulmonary haemodynamics

The pulmonary arterial pressure was measured by introducing a small catheter (polyethylene tubing, ID 0.88 mm, Portex, Hythe, GB) into the pulmonary artery via the jugular vein before the animals were sacrificed. Since the diastolic pulmonary arterial pressure (Pap_D_) reflects the left atrial pressure and thus the end-diastolic left ventricular pressure, we used this parameter to express the haemodynamic consequence of a LVD. Because of the technical demands of this manoeuvre for successful introduction of the miniature catheter in rats, this measurement proved technically acceptable only in subgroups of the main study groups, involving 7, 6, 7 and 10 rats in Groups C, I, ID and IE, respectively.

### Measurement protocol and bronchoprovocation challenges

The experimental protocol included the follow-up of the same animals through the 8-week study period by performing the identical procedure at weeks 0 and 8 (Figure 1). On each occasion, the rats were anaesthetized and prepared as detailed above. EELV was first measured while the rats were breathing spontaneously. A femoral vein was then cannulated for drug delivery. When stable haemodynamic and respiratory mechanical conditions had been reached, we recorded a set of Zrs data including the collection of 4-6 6-s data epochs under the baseline conditions. Increasing doses of iv methacholine (MCh) were then administered at doses of 2, 6 and 18 μg/kg/min. 3-5 Zrs recordings were made at each infusion rate during the steady-state bronchoconstriction that developed, usually 3-5 min after the onset of MCh. Following these measurements, Pap_D_ was measured as described above.

### Statistical analyses

The scatters in the parameters were expressed by the SD values. The Kolmogorov-Smirnov test was used to check data for normality. Two-way repeated measures analysis of variances (ANOVA), with variables of treatment (i.e. the protocol groups) and measurement condition (i.e. weeks 0 and 8), was used to evaluate the effects of the various types of medication on the respiratory mechanical parameters and EELV and the alterations in lung responsiveness to the constrictor challenge. The pulmonary haemodynamic differences between the groups were tested by using one-way ANOVA. Pairwise comparisons were performed by means of Student-Newman-Keuls multiple comparison procedures. The Pearson correlation test was used to assess the strength of associations between variables. Statistical tests were carried out with the significance level set at p < 0.05.

## Results

There was no significant difference in body weight between the protocol groups at the beginning of the study (ranging from 308 ± 56 g to 314 ± 62 g, p = 0.71). Eight weeks later, however, the body weight of the animals in Group IE (463 ± 9 g) was significantly lower than those of the rats in Groups C (518 ± 27 g, p < 0.001), I (498 ± 32 g, p = 0.014) or ID (498 ± 30 g, p = 0.014). The changes in the breathing frequency during EELV measurements reflected these developmental changes with decreases from 66 ± 22, 74 ± 13, 78 ± 20 and 74 ± 16 breath/min at week 0 (p = 0.6) to 39 ± 8, 44 ± 14, 43 ± 18 and 54 ± 23 breath/min at week 8 (p = 0.29) in Groups C, I, ID and IE, respectively.

**Figure 2:**
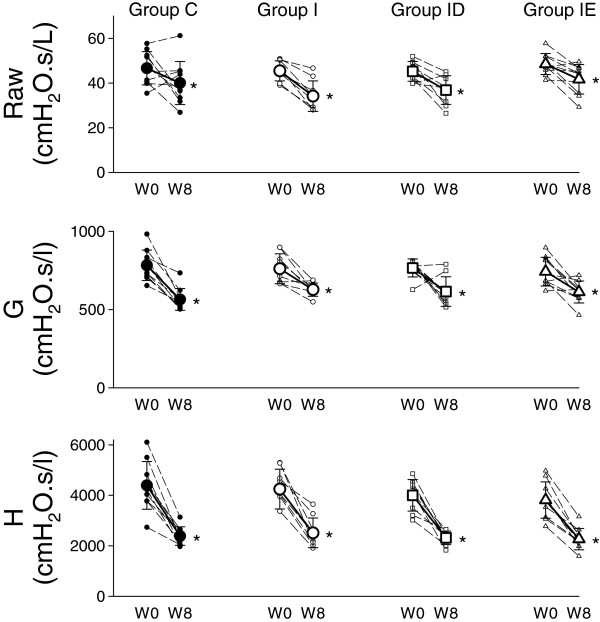
**Changes in the baseline values of the respiratory mechanical parameters obtained by forced oscillation before (W0) and 8 weeks after the induction of a LVD (W8) in rats that underwent sham surgery (Group C), and in rats with a LVD without subsequent treatment (Group I) or supplemented with treatment with calcium channel (Group ID) or ACE blockers (Group IE).** Raw: airway resistance, G: tissue damping, H: tissue elastance. Thin dashed lines with small symbols: individual animals; thick lines with large symbols: group means. *: p < 0.05 W0 vs. W8.

The baseline values of the airway and respiratory tissue mechanical parameters exhibited significant decreases (p < 0.001 for all) between the two study occasions (Figure 2). We found that none of the parameters displayed a significant difference between the study groups under the baseline condition (p = 0.16, 0.87 and 0.38 for Raw, G and H, respectively). The lack of any significant interaction between the measurement time and the protocol group demonstrated that the induction of the LVD and its treatments had no significant effects on the changes in the mechanical parameters (p = 0.52, 0.25 and 0.54 for Raw, G and H, respectively).

**Figure 3:**
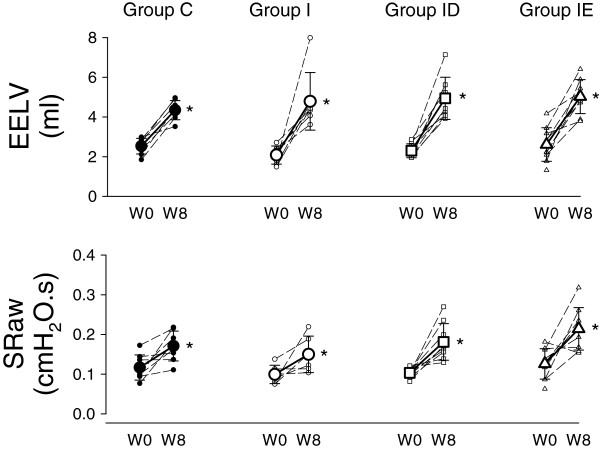
**Changes in the end-expiratory lung volume (EELV), its value normalized to body weight (Normalized EELV) and the specific airway resistance (SRaw) before (W0) and 8 weeks after LVD induction (W8) in rats that underwent sham surgery (Group C), and in rats with a LVD without subsequent treatment (Group I) or supplemented with treatment with calcium channel (Group ID) or ACE blockers (Group IE).** Thin dashed lines with small symbols: individual animals; thick lines with large symbols: group means. *: p < 0.05 W0 vs. W8.

EELV and the baseline SRaw increased significantly (p < 0.001 for both) in all groups by the end of the 8-week study period (Figure 3). The percentage relative increase in EELV after 8 weeks was significantly higher in the Group ID (118 ± 48%) thank in Group C (75 ± 30%, p = 0.048), and there was a tendency for an excessive EELV increase in group I (145 ± 107%, p = 0.09). Mild but statistically significantly more pronounced elevation in SRaw were observed in the rats of Group IE (p = 0.047) by the end of the 8-week study period (Figure 3).

**Figure 4:**
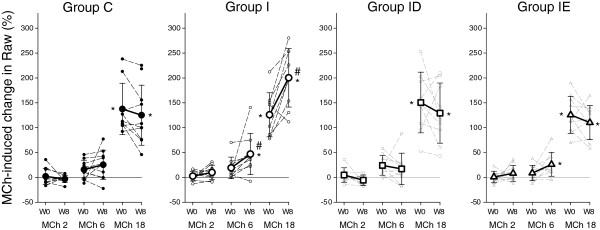
**Alterations in lung responsiveness expressed as changes in airway resistance during increasing doses of methacholine (MCh 2, 6 and 18 μg/kg/min).** Data points were obtained before (W0) and 8 weeks after LVD induction (W8) in rats that underwent sham surgery (Group C), and in rats with a LVD without subsequent treatment (**Group I**) or supplemented with treatment with calcium channel (**Group ID**) or ACE blockers (**Group IE**). Thin dashed lines with small symbols: individual animals; thick lines with large symbols: group means. *: p < 0.05 W0 vs. W8; #: p < 0.05 vs. the corresponding data point obtained in Group C.

MCh provocations induced a dose-dependent bronchoconstriction in all groups (p < 0.001, Figure 4). No change in airway responsiveness was detected in Group C (p = 0.7), whereas the induction of a LVD led to the development of BHR in the rats in Group I (p = 0.02), as illustrated by the significant upward shift in the dose–response curve. Each of the treatment strategies exerted a protective effect against this hyperresponsiveness to MCh, with abolition of the difference in airway responsiveness in Groups ID (p = 0.3 week 0 vs. week 8) and IE (p = 0.65 week 0 vs. week 8). These findings were reflected in the lack of significant change in ED_50_ in Groups C (9.2 ± 1.9 vs. 10.2 ± 4.4 μg/kg/min, p = 0.65), ID (9.5 ± 3.6 vs. 10.7 ± 4.7 μg/kg/min, p = 0.33) and IE (10.6 ± 2.1 vs. 9.8 ± 3.5 μg/kg/min, p = 0.56), whereas it decreased markedly in the rats in Group I (10.0 ± 2.5 vs. 6.9 ± 2.5 μg/kg/min, p = 0.006).

**Figure 5:**
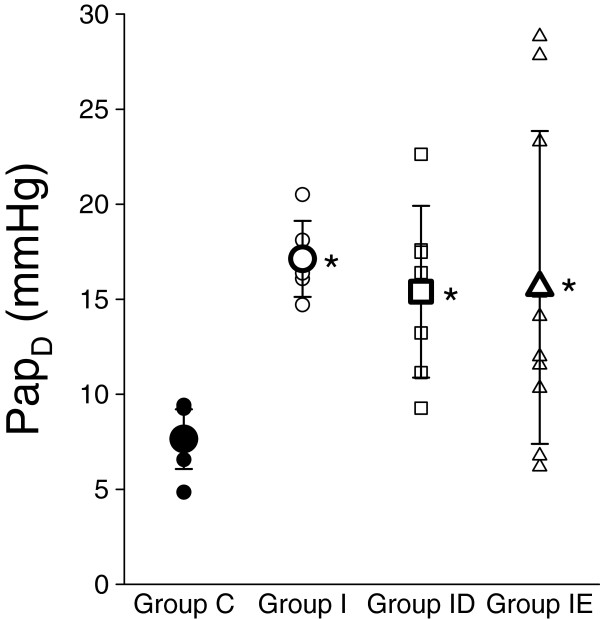
**Diastolic pulmonary arterial pressures in the rats involved in the different groups.Group C:** control group that underwent sham surgery, **Group I:** rats with a LVD without further treatment, **Group ID:** rats with a LVD treated with a calcium channel blocker (diltiazem). **Group IE:** rats with a LVD treated with an ACE blocker (enelapril). *: p < 0.05 vs. the data obtained in Group C.

The induction of the LVD increased Ppa_D_ in all groups where pulmonary hypertension was generated by coronary ischaemia (Figure 5), with variable but significant elevations in Groups I (p = 0.004), ID (p = 0.013) and IE (p = 0.006).

**Figure 6:**
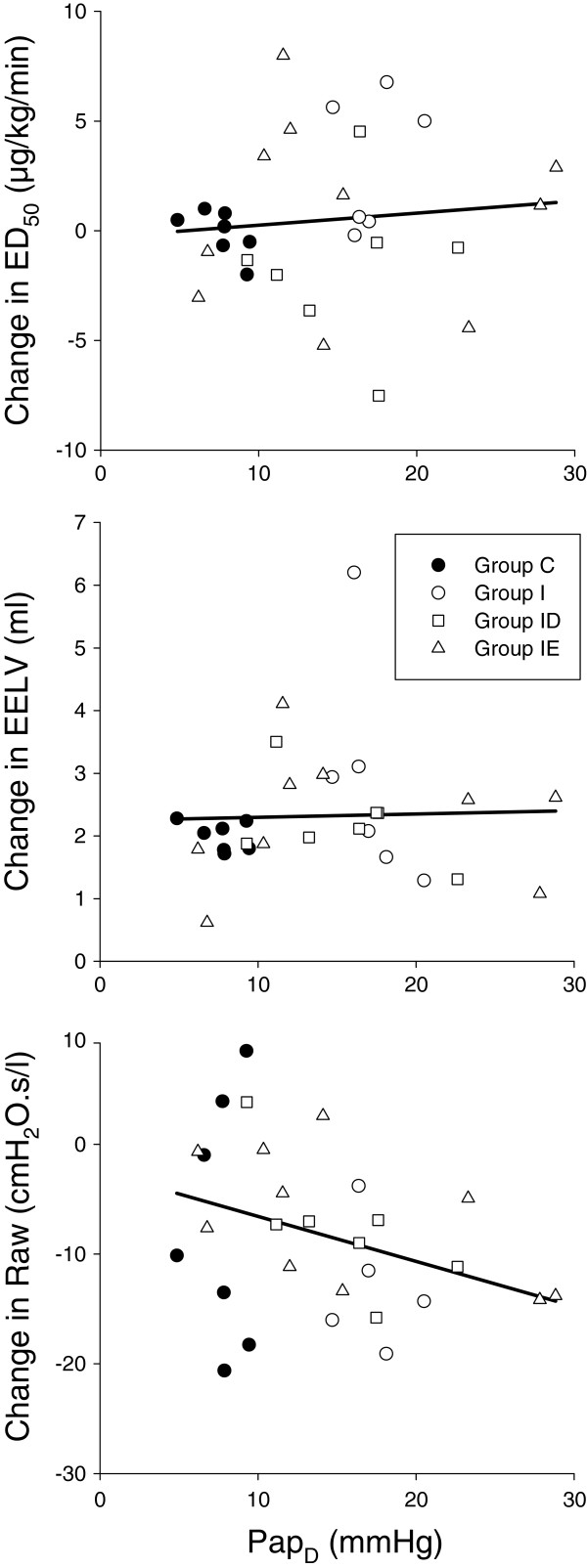
**Relationships between the diastolic pulmonary arterial pressures (Ppa**_**D**_**) measured 8 weeks after LVD induction and the changes in lung responsiveness to MCh (ED**_**50**_**) in the end-expiratory lung volume (EELV) and in the airway resistance (Raw).** Data points were obtained in rats that underwent sham surgery (Group C), and in rats with a LVD without subsequent treatment (Group I) or supplemented with treatment with calcium channel (Group ID) or ACE blockers (Group IE).

The analyses of the associations between the changes in pulmonary haemodynamics and respiratory mechanics revealed no significant correlations between the magnitude of pulmonary haemodynamic changes expressed as Pap_D_ and the alterations in lung responsiveness expressed as the difference in ED_50_ between weeks 0 and 8 (R = 0.097, p = 0.61, Figure 6). Furthermore, no significant correlation was observed between Pap_D_ and the 8-week changes in EELV (R = 0.033, p = 0.86), while there was a tendency to a strong association between Pap_D_ and the 8-week changes in Raw (R = -0.35, p = 0.06).

## Discussion

Various treatment strategies are applied in the presence of a LVD in order to enhance the cardiac output by optimizing the preload of the left ventricle and subsequently decreasing the pulmonary vascular engorgement or by decreasing the systemic vascular resistance. In the present study, we investigated the abilities of treatments considered against a LVD to protect the potentially adverse pulmonary consequences of left heart failure. We applied a well-validated animal model of coronary ischaemia to induce a sustained elevation of left atrial pressure, leading to postcapillary PHT and the subsequent development of BHR. Under this pathophysiological condition, both treatment strategies (based on blockade either of the rennin-angiotensin pathway supplemented with diuretics or of the calcium channels) abolished the enhanced lung responsiveness to exogenous constrictor stimuli without affecting the lung volume increase. Examination of the relationships between the pulmonary haemodynamic and lung functional changes revealed that the diastolic Pap level and the magnitude of the lung responsiveness were dissociated.

In agreement with the earlier reported findings [11,18], the induction of left ventricular myocardial ischaemia led consistently to postcapillary PHT, as proved by the marked elevations in Pap_D_ in the present study. The achievement of more detailed experimentation to characterize the altered pulmonary haemodynamics in the present study was complicated by the small size of the model animal and the lack of a catheter-tip balloon facilitating its introduction into the pulmonary artery. This technical drawback was the main reason for the availability of a technically acceptable Pap_D_ recording in only about two-thirds of the rats, which agrees well with the previous success rate in this species when a similar technique was used [11,19]. Due to the technically challenging nature of the surgical preparation of LVD, one control group of rats with LVD was assessed (Group I) instead of involving separate control groups of rats with ip saline injections or oral placebo treatment. Since the ip (Group ID) and oral (Group IE) treatments of LVD necessitated only mild restrains of the animals for short period of times, the interventions related to the treatments *per se* were not likely to bias our findings.

In the presence of a LVD, the systematic enhancement of the airway responsiveness to exogenous constrictor stimuli as a hallmark feature of BHR was confirmed in the present study [11]. In complete agreement with a previous observation [20], there was no significant correlation between the degree of lung congestion estimated from Pap_D_ and the severity of BHR (Figure 6), confirming that a more severe pulmonary vascular congestion after a LVD does not necessarily involve a more severe BHR. This consistent finding demonstrates that the enhanced MCh responsiveness following a sustained LVD is not directly related to the altered geometry of the congested pulmonary vessels. Rather, LVD may trigger a cascade mechanism via a release of numerous broncho- and vasoactive mediators, causing the development of vascular remodelling and subsequent BHR.

It is noteworthy that BHR was observed previously on use of a constrictor challenge with serotonin [11] and this feature was fully reproduced in the present study by applying MCh as a provocation agonist. Since serotonin and MCh induce bronchoconstriction by activating different receptors, with the former acting on the muscarinic and the latter stimulating the 5-hydroxytryptamine receptors, the consistent detection of BHR demonstrates that, in the presence of a sustained LVD, the enhanced lung responsiveness is independent of the receptor subtype via which the different agonists exert their bronchoconstrictive potentials.

While all respiratory mechanical parameters at the baseline level exhibited marked decreases in the 8-week study period, due to the somatic growth (Figure 2), neither the presence of the LVD nor the treatments affected them significantly. This finding agrees with the previous experimental data indicative of a lack of change in the baseline values of airway or respiratory tissue mechanics following a sustained pre- [19,21] or postcapillary PHT [11]. Furthermore, this result is in line with previous clinical observations that demonstrated no significant effects of a LVD on the basal airway [22] or respiratory [23] resistance. The present study is the first to demonstrate a follow-up in the lung volume changes before and after a sustained LVD. The changes observed in EELV and in the breathing rate during the 8-week study period can be explained almost fully by the lung growth (Figure 3, top). Since the EELV values were corrected for the thermal characteristics of the plethysmograph, the uniform alterations in the breathing rate in the protocol groups was not likely to bias our estimates [13]. The strong tendency for excessive increases in the EELV in the rats with a LVD may be attributed to the involvement of the autonomous nervous system in the presence of hypoxia [12,24] that might have been present after the induction of LVD [1].

The use of calcium channel blockers in the presence of LVD has been challenged in clinical practice in the past decade [25]. However, among the calcium antagonists, diltiazem was demonstrated to exhibit the best profile without a significant negative inotropic effect and an improvement in cardiac function following coronary ischaemia [26]. Our findings suggest that diltiazem did not inhibit the pulmonary vascular engorgement, as revealed by the preservation of a high Pap_D_ in the majority of the rats treated for 8 weeks (Figure 5). Conversely, diltiazem prevented the development of BHR (Figure 4) by diminishing the bronchoconstriction at all doses of MCh. This beneficial effect is most probably due to the effect of diltiazem on the calcium channels, with a consequent decrease in the Ca^2+^ influx into the smooth muscle cells. Since Ca^2+^ is essential for the contractility of the airway smooth muscle cell, diltiazem may exert its potential protection effect via this cellular mechanism [27].

A LVD following coronary ischaemia leads to a decrease in cardiac output and stimulates the renin-angiotensin-aldosterone pathway, which then results in renal sodium and water retention, contributing further to the overload, and hence increasing the preload of the left ventricle [28,29]. A combination of enalapril and diuretics is therefore the first-line treatment of cardiac failure, through inhibition of this mechanism, leading to optimization of the preload of the left ventricle and a subsequent decrease in the pulmonary venous pressure. In agreement with the literature, the 8-week treatment with enalapril slightly retarded the body weight gain most probably by affecting the metabolic homeostasis [30]. Furthermore, this treatment proved to be beneficial in the majority of animals, with a decrease in Pap_D_, though some rats still exhibited a very high pulmonary venous pressure (Figure 5). Despite this fairly diverse pulmonary vascular effect, enalapril exhibited a uniform beneficial effect on the respiratory mechanics and airway responsiveness. It is noteworthy that this treatment slightly but statistically significantly elevated the baseline value of SRaw, which may be related to the irritating properties of enalapril on the airways, occasionally causing mild airway obstruction [31]. As regards the potential of ACE inhibition to affect BHR, there have been conflicting findings, with some authors promoting its beneficial profile [32,33], others reporting no change in lung reactivity [34] and some even demonstrating a worsening airway responsiveness [35,36]. Our results are in line with those suggesting that ACE inhibition with enalapril may counterbalance the constrictor effect of angiotensin on the airway smooth muscle and may blunt the BHR observed following cholinergic stimulation with MCh [32]. Since the level of angiotensin is expected to increase following a LVD, the blockade of this pathway may be responsible for this beneficial pulmonary profile observed in the present study. Besides this potential beneficial profile of enalapril, the supplemental administration of furosemide may further contribute to the inhibition of BHR, since this diuretics was proved to decrease airway responsiveness in humans [37] and in rodents [38]. This effect may be related to ease of the mechanical load exerted by the congested pulmonary capillaries on the airways and/or to the modulation of the neurotransmission [37].

### Summary and conclusions

Surgical induction of coronary ischaemia in the present study confirmed that a sustained increase in left atrial pressure and subsequently in the pulmonary vasculature enhances the airway responsiveness to exogenous constrictor stimuli and suggested an elevation of static lung volume. The pathogenesis of this phenomenon was effectively prevented by blocking the calcium channels following the insult and by treating the adverse neurohumoral consequences of the LVD by counteracting the adverse vascular effect of the angiotensin pathway. Relating the efficiency of these treatments on the lung responsiveness and the pulmonary haemodynamics revealed that both strategies exhibit more consistent benefit against airway hyperresponsiveness due to pulmonary vascular engorgement than on the rather variable profile on the lung vasculature. The lack of a close association between the severity of BHR and the degree of lung congestion suggests that broncho- and vasoactive mediators were involved rather than alterations in the geometry of the congested pulmonary vessels. The abolition of airway symptoms in patients with a LVD following these treatment strategies is therefore anticipated.

## Competing interests

The authors declare that they have no competing interests.

## Authors’ contributions

FP coordinated the various experimental approaches, and contributed to their design and to the manuscript preparation. GA and EL carried out the experiments and the preliminary data analyses. MB participated in the design of the study and in the interpretation of the experimental findings. WH conducted the design of the study and had a major role in drafting the manuscript. All authors have read and approved the final manuscript.

## References

[B1] Snashall PD Chung KF Airway obstruction and bronchial hyperresponsiveness in left ventricular failure and mitral stenosis Am Rev Respir Dis 1991 144 945 956 10.1164/ajrccm/144.4.945 1928973

[B2] Gehlbach BK Geppert E The pulmonary manifestations of left heart failure Chest 2004 125 669 682 10.1378/chest.125.2.669 14769751

[B3] Albu G Peták F Fontao F Biton C Pache JC Habre W Mechanisms of airway hyper-responsiveness after coronary ischemia Respir Physiol Neurobiol 2008 162 176 183 10.1016/j.resp.2008.06.009 18602501

[B4] Cabanes LR Weber SN Matran R Regnard J Richard MO Degeorges ME Lockhart A Bronchial hyperresponsiveness to methacholine in patients with impaired left ventricular function N Engl J Med 1989 320 1317 1322 10.1056/NEJM198905183202005 2541334

[B5] Rolla G Bucca C Caria E Scappaticci E Baldi S Bronchial responsiveness in patients with mitral valve disease Eur Respir J 1990 3 127 131 2178962

[B6] Wetzel RC Herold CJ Zerhouni EA Robotham JL Intravascular volume loading reversibly decreases airway cross-sectional area Chest 1993 103 865 870 10.1378/chest.103.3.865 8449083

[B7] Haworth SG Hall SM Panja M Peripheral pulmonary vascular and airway abnormalities in adolescents with rheumatic mitral stenosis Int J Cardiol 1988 18 405 416 10.1016/0167-5273(88)90059-9 3360524

[B8] Chatterjee K Congestive heart failure: what should be the initial therapy and why? Am J Cardiovasc Drugs 2002 2 1 6 10.2165/00129784-200202010-00001 14727993

[B9] Henry PD Comparative pharmacology of calcium antagonists: nifedipine, verapamil and diltiazem Am J Cardiol 1980 46 1047 1058 10.1016/0002-9149(80)90366-5 6255787

[B10] Zema MJ Perlmutter S Mankes S Nikitopoulos C Diltiazem treatment for the management of ischaemia in patients with poor left ventricular function: safety of long term administration Br Heart J 1987 58 512 517 10.1136/hrt.58.5.512 3676040 PMC1277349

[B11] Albu G Petak F Fontao F Biton C Pache JC Habre W Mechanisms of airway hyper-responsiveness after coronary ischemia Respir Physiol Neurobiol 2008 162 176 183 10.1016/j.resp.2008.06.009 18602501

[B12] Habre W Janosi TZ Fontao F Meyers C Albu G Pache JC Petak F Mechanisms for lung function impairment and airway hyperresponsiveness following chronic hypoxia in rats Am J Physiol Lung Cell Mol Physiol 2010 298 L607 L614 10.1152/ajplung.00222.2009 20139180

[B13] Janosi TZ Adamicza A Zosky GR Asztalos T Sly PD Hantos Z Plethysmographic estimation of thoracic gas volume in apneic mice J Appl Physiol 2006 101 454 459 10.1152/japplphysiol.00011.2006 16645196

[B14] Petak F Hantos Z Adamicza A Asztalos T Sly PD Methacholine-induced bronchoconstriction in rats: effects of intravenous vs. aerosol delivery J Appl Physiol 1997 82 1479 1487 10.1063/1.365927 9134896

[B15] Petak F Habre W Hantos Z Sly PD Morel DR Effects of pulmonary vascular pressures and flow on airway and parenchymal mechanics in isolated rat lungs J Appl Physiol 2002 92 169 178 11744657 10.1152/jappl.2002.92.1.169

[B16] Hantos Z Daroczy B Suki B Nagy S Fredberg JJ Input impedance and peripheral inhomogeneity of dog lungs J Appl Physiol 1992 72 168 178 10.1063/1.352153 1537711

[B17] Petak F Hall GL Sly PD Repeated measurements of airway and parenchymal mechanics in rats by using low-frequency oscillations J Appl Physiol 1998 84 1680 1686 10.1063/1.368257 9572817

[B18] Jasmin JF Calderone A Leung TK Villeneuve L Dupuis J Lung structural remodeling and pulmonary hypertension after myocardial infarction: complete reversal with irbesartan Cardiovasc Res 2003 58 621 631 10.1016/S0008-6363(03)00290-6 12798435

[B19] Habre W Albu G Janosi TZ Fontao F von Ungern-Sternberg BS Beghetti M Petak F Prevention of bronchial hyperreactivity in a rat model of precapillary pulmonary hypertension Respir Res 2011 12 58 10.1186/1465-9921-12-58 21524300 PMC3096918

[B20] Sasaki F Ishizaki T Mifune J Fujimura M Nishioka S Miyabo S Bronchial hyperresponsiveness in patients with chronic congestive heart failure Chest 1990 97 534 538 10.1378/chest.97.3.534 2306955

[B21] von Ungern-Sternberg BS Habre W Regli A Pache JC Fontao F Janosi TZ Beghetti M Petak F Precapillary pulmonary hypertension leads to reversible bronchial hyperreactivity in rats Exp Lung Res 2010 36 129 139 10.3109/01902140903214667 20334605

[B22] Yap JC Moore DM Cleland JG Pride NB Effect of supine posture on respiratory mechanics in chronic left ventricular failure Am J Respir Crit Care Med 2000 162 1285 1291 11029332 10.1164/ajrccm.162.4.9911097

[B23] Spyratos DG Glattki GP Sichletidis LT Patakas D Assessment of respiratory mechanics by impulse oscillometry in orthopneic patients with acute left ventricular failure Heart Lung 2011 40 97 104 10.1016/j.hrtlng.2010.05.055 20723981

[B24] Vizek M Frydrychova M Houstek S Palecek F The role of vagal nerves in changes of functional residual lung capacity during acute hypoxia Physiol Bohemoslov 1983 32 318 323 6622556

[B25] Boden WE van Gilst WH Scheldewaert RG Starkey IR Carlier MF Julian DG Whitehead A Bertrand ME Col JJ Pedersen OL Diltiazem in acute myocardial infarction treated with thrombolytic agents: a randomised placebo-controlled trial. Incomplete Infarction Trial of European Research Collaborators Evaluating Prognosis post-Thrombolysis (INTERCEPT) Lancet 2000 355 1751 1756 10.1016/S0140-6736(00)02262-5 10832825

[B26] Follath F The role of calcium antagonists in the treatment of myocardial ischemia Am Heart J 1989 118 1093 1096 discussion 1096-1097 10.1016/0002-8703(89)90832-6 2683702

[B27] Triggle DJ Calcium, the control of smooth muscle function and bronchial hyperreactivity Allergy 1983 38 1 9 10.1111/j.1398-9995.1983.tb00849.x 6301306

[B28] Dube P Weber KT Congestive heart failure: pathophysiologic consequences of neurohormonal activation and the potential for recovery: part I Am J Med Sci 2011 342 348 351 10.1097/MAJ.0b013e318232750d 22030844

[B29] Dube P Weber KT Congestive heart failure: pathophysiologic consequences of neurohormonal activation and the potential for recovery: part II Am J Med Sci 2011 342 503 506 10.1097/MAJ.0b013e3182327527 22108172

[B30] Santos EL de Picoli Souza K da Silva ED Batista EC Martins PJ D'Almeida V Pesquero JB Long term treatment with ACE inhibitor enalapril decreases body weight gain and increases life span in rats Biochem Pharmacol 2009 78 951 958 10.1016/j.bcp.2009.06.018 19549507

[B31] Lunde H Hedner T Samuelsson O Lotvall J Andren L Lindholm L Wiholm BE Dyspnoea, asthma, and bronchospasm in relation to treatment with angiotensin converting enzyme inhibitors BMJ 1994 308 18 21 10.1136/bmj.308.6920.18 8298346 PMC2539116

[B32] Rubini A Redaelli M Parmagnani A The effect of angiotensin-converting enzyme inhibition by captopril on respiratory mechanics in healthy rats J Enzyme Inhib Med Chem 2012 27 6 854 860 10.3109/14756366.2011.622271 22591316

[B33] Watanabe K Myou S Fujimura M Tachibana H Kita T Nakao S Importance of the angiotensin type 1 receptor in angiotensin II-induced bronchoconstriction and bronchial hyperresponsiveness in the guinea pig Exp Lung Res 2004 30 207 221 10.1080/01902140490276366 15195554

[B34] Kaufman J Schmitt S Barnard J Busse W Angiotensin-converting enzyme inhibitors in patients with bronchial responsiveness and asthma Chest 1992 101 922 925 10.1378/chest.101.4.922 1555463

[B35] Matsubara S Kikkawa H Kaminuma O Ikezawa K Angiotensin-converting enzyme inhibitors can potentiate ozone-induced airway hyperresponsiveness Eur J Pharmacol 1997 337 259 265 10.1016/S0014-2999(97)01316-2 9430423

[B36] Bucknall CE Neilly JB Carter R Stevenson RD Semple PF Bronchial hyperreactivity in patients who cough after receiving angiotensin converting enzyme inhibitors Br Med J (Clin Res Ed) 1988 296 86 88 10.1136/bmj.296.6615.86 PMC2544699 2827838

[B37] Crimi N Prosperini G Ciamarra I Mastruzzo C Magri S Polosa R Changes in neurokinin A (NKA) airway responsiveness with inhaled frusemide in asthma Thorax 1997 52 775 779 10.1136/thx.52.9.775 9371207 PMC1758644

[B38] Wang S Xiang YY Ellis R Wattie J Feng M Inman MD Lu WY Effects of furosemide on allergic asthmatic responses in mice Clin Exp Allergy 2011 41 1456 1467 10.1111/j.1365-2222.2011.03811.x 21729180

